# Topics of Public Interest

**Published:** 1910-04

**Authors:** 


### TOPICS OF PUBLIC INTEREST.

### Common Colds

**Common Colds might easily be made uncommon, says physician—Open meeting at New York Academy of Medicine, March 24, 1910.**

(*Tribune*, March 25, 1910)

COMMON colds would become one of the most uncommon things in the world if people would wash out their nasal passages properly night and morning. So, at least, Dr. Abraham Jacobi and several other physicians assured a large audience at the Academy of Medicine, Thursday afternoon, March 24, 1910. Dr. William Kelly Simpson brought along a pretty schoolgirl in a sailor suit, on whom he made a practical demonstration to show how easy it is to give one's self the right sort of nose douche and ear douche.

One cause of colds in children, Dr. Jacobi said, was the practice of putting them in short socks and leaving the calves of their legs bare. "This is done," he said, "not by women, but by ladies," who ought not to have any children—and frequently the undertaker gets the children they have."

Dr. Jacobi said he took a cold plunge 365 times a year, and was sure he should have died twenty times of pneumonia or some other "providential" disease without it. Still, he doesn't recommend cold baths for every one.

"A cold plunge is too severe for the anemic, the very young or the old," said Dr. Jacobi, who is close on fourscore himself.

"The direct cause of a cold is bacteria," said Dr. Emily Lewi. "The indirect causes are all the many things that may upset the equilibrium. It may be a lobster supper, it may be a great mental strain or sorrow—anything that congests the bloodvessels in any part of the body and stops the throwing off of the poisonous secretions. We eat too much, we eat too fast, we eat under a nervous strain, we do too many things directly after eating. The mucous membrane congests and the bacteria get in their work."

Dr. Mary MacMillan said that her hobby was wet feet. "If it were practicable," she added, "for every child to keep a pair of shoes at school and change whenever he reached school with damp shoes, the number of colds would be fewer. It is well to wear light woolen underwear, but many people cannot bear it next the skin, and with our overheated houses it is becoming more and more the practice to wear thin underwear and depend on warm wraps when going out of doors.

"Muffling the neck in furs is a common cause of colds among women, and so is the custom of wearing a high collar all day and putting on a décolleté gown at night."

Dr. Simpson's talk, illustrated by the nasal douche, dealt with the treatment of the passages of the head. Dr. Jacobi showed the nose douche he used. which was much simpler than Dr. Simpson's. In closing, Dr. Jacobi said that underfed, depleted and overworked people were much more liable to colds than the well cared for. "The neglected poor," he said, "are thus a menace, not only to themselves but to their richer neighbors—a source of dissemination of disease."

The lecture was one of the course arranged by the public health education committee of the County Medical Society and the hygiene committee of the City Federation of Women's Clubs.

---

### Bureau of the Census

**The Bureau of the Census Explains—Wrong views of the Census—No harm can come to any person who answers the questions.**

DEPARTMENT OF COMMERCE AND LABOR,

BUREAU OF THE CENSUS.

Washington, D. C., March 2, 1910.

Letters from the census supervisors to the United States Census Bureau show the erroneous apprehension of a considerable element of the population that their answers to the enumerators' questions in the next census, beginning April 15, this year, will cause increased taxation, legal entanglements, or injurious consequences to their persons and property.

In order to quiet such unfounded fears, which would, unless removed, materially affect the accuracy of the census, the bureau has prepared an official statement relative to the decennial census, its origin, purpose, and uses.

This statement should furnish complete assurance to those concerned that information given the enumerators is held by the Census Bureau in the strictest confidence with reference to the identity of the informants, as required by the policy of the bureau and commanded by the law of the United States.

The bureau earnestly hopes that clergymen, priests, physicians, school-teachers, employers, and other public-spirited citizens who come in contact with large numbers of people, will co-operate with the bureau by telling persons who are believed to entertain erroneous opinions of the census the real facts and urging them to give full replies to the enumerators. Teachers are particularly requested to speak of the census to the school children and ask them to tell their parents about it.

The statement issued by the bureau explains that the Constitution requires a census of the population to be taken every ten years in order to reapportion state representation in the National House of Representatives. It is the means also to ascertain the increase in the population, agriculture, industries, and resources of the nation since the last census.

It is emphatically declared, by the statement, that the information sought from the people of the United States is used solely for general statistical purposes. It will neither be published nor used in any other way to disclose facts regarding any individual or enterprise. The census, it goes on to say, is not, never has been, and can not be employed to obtain information that can be used in any way in the assessment of property for purposes of taxation or the collection of taxes, either national, state, or local; or for deportation proceedings, extradition measures, army or navy conscription, internal-revenue investigations, compulsory school attendance, child-labor law prosecutions, quarantine regulations, or in any way to affect the life, liberty, or property of any person.

It points out that replies to the enumerators are and must be held by the Census Bureau in strict and absolute confidence. All the bureau officials, supervisors, supervisors' clerks, enumerators, and interpreters, before entering upon their duties, are obliged to take a solemn oath not to disclose any information they may obtain except to the Census Bureau, and a violation of the United States law in regard to this oath means a $1,000 fine or imprisonnment for two years, or both, in the discretion of the court.

### No Uncared-for Tuberculosis in 1915

**Conference of Consumption Fighters Opens—Mayor Duryee Wants Law Enforced—Doctors are Negligent—500 in Attendance.**

Delegates to the Conference of the Local Committees on the Prevention of Tuberculosis of the State Charities Aid Association, which opened at Albany, March 18, 1910, were greeted at every turn with the cry, "No Uncared-for Tuberculosis in 1915." From the opening remarks of Honorable Homer Folks, to the last bit of discussion on the enforcement of the tuberculosis laws requiring registration, to say nothing of the big placard hung in the front of the hall, and the programs, in every speech and at every corner the determination to provide for every tuberculosis case in the next five years was manifest.

Delegates, numbering over 500, were in attendance at the Conference, representing all sections of the state, besides many visitors from adjoining states. President Taft and Governor Hughes addressed the meeting on the nineteenth.

Honorable Homer Folks, Secretary of the State Charities Aid Association, opened the Conference with emphasis on the fact that the gathering had been called to promulgate a definite ideal for 1915. He said in part:

Some conferences are just plain conferences; and others have a precise, definite purpose. This conference which I now have the pleasure of calling to order is of the latter sort. A very definite, specific purpose led to the calling of the conference and gives a principle of unity and continuity to all its sessions. That purpose is to consider and adopt a definite line of action in our tuberculosis campaign for the next five years.

From the point of vantage which we have now gained from our position of some elevation, we can look forward over the next four or five years; we can see in the distance all the chief positions of the enemy which remain to be captured; and we have met here for the purpose of outlining the position of our forces, the various movements which must be made in consecutive order and in relation to the others, and which we can now say with the certainty of a Japanese general commanding his forces in the field, will result in five years' time in the capture of all the territory now spread out before us. The goal of our efforts, the sweep of the territory which we most fully occupy, is summed up in the phrase "No Uncared-for Tuberculosis in 1915." The content of that brief phrase is set forth in the program of this meeting, and will, we hope, be placed before each of you not once but many times through the press and by other agencies of publicity.

The one point which I wish to emphasise at the moment is that that phrase expresses not a hope, but a purpose; not a vague anticipation, but a deliberate determination; not an impracticable scheme, but a perfectly practicable undertaking; a thing well within the limits of things that can be done without interfering with other things that ought to be done; a thing which can be accomplished, and accomplished within the period set. Such, in a word, is the keynote of this conference. All the papers to be presented, all the addresses to be made, are expected simply to give content to this expression, and to lend urgency to its plea.

Mr. Folks then announced the topic of the first session, "Discovery and Supervision of Cases in the Home," and introduced Hon. Charles C. Duryee, Mayor of Schenectady, as the first speaker on the subject, "Enforcement of the Tuberculosis Law, the Duty and Opportunity of the Local Health Officer." Mayor Duryee is a physician and was formerly health officer of his city. He made his winning campaign for mayor on the platform of tuberculosis and its prevention.

Dr. Duryee took as his theme Chapter 351 of the Laws of 1908, which requires that tuberculosis cases should be reported and looked after by local health officers, physicians and others. He said of this law:

It removed tuberculosis, one of the most prevalent and widely scattered of all diseases, from the range of what we may call personal and individual responsibility, to that of social responsibility, and made the health officer the chief factor in the enforcement of that social responsibility.

Dr. Duryee argued that the health officer should realise this responsibility and that he should see that every tuberculosis case reported to him is properly cared for, either at home or elsewhere, and gave several experiences concerning the enforcement of the law in Schenectady.

In conclusion, Dr. Duryee said:

I would most cordially recommend to all health officers that, distasteful as they may find the close study of legal phraseology, they make the reading and re-reading of Chapter 351, Laws of 1908, now to be found as Sections 320-331 inclusive, Chapter 45 of the Consolidated Laws of 1909, a part of their routine from week to week. New horizons will be opened from the re-reading of this comprehensive statute, and only by its re-examination in the light of actual experience in its enforcement will its full sweep and potential effect upon sanitary uplift of this state become evident. A copy of this statute should take its place along with the Manual of Laws and the Family Bible, in the house of the health officer. The same advice might modestly be given to every physician in the state. Health officers cannot do better than to send copies of this law in its entirety from time to time to all the physicians of their localities.

Mayor Duryee's paper was followed by a discussion and a Symposium on "Agencies for Carrying into Effect the Provisions of the Law." Dr. Henry L. K. Shaw, Secretary of the Albany Committee on the Prevention of Tuberculosis, gave a heart-to-heart talk on what the physician could do. He emphasized the fact that it was the moral obligation of every physician to obey the tuberculosis law just as he did other laws, and that such a view of the situation would greatly increase the number of cases reported. He showed also how, in failing to report the cases of tuberculosis which came to his knowledge, the physician was blocking the entire tuberculosis program of provision for the consumptive, since without knowledge of the whereabouts of tuberculosis cases, agencies for the control, relief or prevention of this disease could do little.

Dr. H. W. Carey, Secretary of the Troy Tuberculosis Relief Committee, told of the value of the dispensary as a means for discovering early cases and for following up cases that had already been reported.

Miss Anna Lantz, Visiting Nurse of the Geneva Public Health Committee, spoke of the service of the visiting nurse in carrying out the provisions of the tuberculosis law.

Miss Ethel Van Benthuysen, Chairman of the Relief Committee of the Albany Association, showed how the proper reporting of tuberculosis cases afforded opportunities for relief that could not be secured in any other way, and urged greater co-operation and unity among the agencies giving relief to the consumptive.

In the evening a banquet was served to the delegates at the Hotel Ten Eyck, at which the subject of "Tuberculosis as a School Problem" was discussed. Professor George F. Canfield, Chairman of the State Committee on the Prevention of Tuberculosis, presided, and addresses were given by Dr. Oscar H. Rogers, of Yonkers, on "Teaching the Essential Facts to Children;" by Dr. George W. Goler on "Medical Inspection of School Children with Respect to the Prevention of Tuberculosis;" and by Leonard P. Ayres, of the Department of Child Hygiene of the Russell Sage Foundation, on "Open Air Schools for Children Predisposed to Tuberculosis."

---

"Which way is the business district from here?" the stranger asked.

"I dunno," said the boy.

"Which way shall I go to get down town?"

"I dunno."

"What do you know about the city?"

"We're goin' to have a new stachun—sometime," he said.

### Women's Medical Society of New York

**Dr. L. Duncan Bulkley Lectures on Leprosy—Exhibits Two Lepers Before His Audience—Declares that During His Thirty Years' Practice Has Never Seen Evidence that the Disease is Contagious— Dr. W. S. Bainbridge and Others also Entertain the Women's Medical Society.**

(*New York American*, March 13, 1910.)

Dr. L. Duncan Bulkley, the eminent dermatologist, in a lecture on skin diseases before the Women's Medical Society of New York, at the Skin and Cancer Hospital, March 12, 1910.

Dr. Bulkley brought forward two West Indian lepers who have been patients at the hospital for several months. The disease is well defined in both, one having developed the "leonine countenance" characteristic of a certain stage of leprosy.

"These two patients," said Dr. Bulkley, "are permitted to mingle with the other patients who are not suffering from contagious disease, just as did "Leper" Early, who, I demonstrated, was not suffering from leprosy while he was in the hospital.

The fear of leprosy is purely a superstition and comes down from Biblical times, yet the leprosy of the Bible was not the modern disease that we know. For their own safety the people of Biblical times classed most of the skin diseases and the Hebrew word for such diseases has been wrongly translated as leprosy. Moses was not a physician nor were the priests, and neither knew much of medicine.

I have never hesitated to handle leprosy cases in my office and the hospital just as I would other non-contagious diseases. It is true, however, that we know little of its transmission except that it has been contracted from certain fish which have first been infected, just as typhoid is caught from infected oysters. I have never known of a single case of its being transferred from one person to another."

Dr. Bulkley also produced a peddagra patient of the Skin and Cancer Hospital, who, so far as is known, is the only person suffering from the disease in New York, and demonstrated the symptoms of the disease to the visitors.

About seventy members of the Women's Medical Society were present during the lecture and were entertained at the hospital.

For their benefit Dr. W. S. Bainbridge, of the hospital staff, performed three operations in the skin and cancer clinic, and Dr. J. T. Gawthmey demonstrated his invention of a new method of applying anesthetics, which does away with the delirium and nausea which usually accompany the process. This was followed by a demonstration of *x*-ray and Finsen Light applications by Dr. G. A. Lawrence. The women were also entertained at luncheon at the hospital.

It was announced during the lecture that the hospital was to establish an annex for the care and observation of incurable cancer patients as soon as the necessary funds are raised. The proposed addition will increase the accommodations of the hospital by 30 or 40 per cent.

